# Killer-Cell Immunoglobulin-Like Receptors (KIR) in HIV-Exposed Infants in Cameroon

**DOI:** 10.1155/2021/9053280

**Published:** 2021-01-13

**Authors:** Kagoué Simeni Luc-Aimé, Yindom Louis-Marie, Loni Ekali Gabriel, Clauvis Kunkeng Yengo, F. Esemu Livo, Nguedia Jules Clement Assob

**Affiliations:** ^1^Department of Medical Laboratory Sciences, Faculty of Health Sciences, University of Buea, Buea, Cameroon; ^2^Nuffield Department of Medicine, University of Oxford, Oxford, UK; ^3^National Aids Control Committee, Yaoundé, Cameroon; ^4^Department of Biochemistry, Faculty of Sciences, University of Yaoundé I, Yaoundé, Cameroon; ^5^Institute of Medical Research and Medicinal Plant Studies, Ministry of Scientific Research and Innovation, Yaoundé, Cameroon

## Abstract

The biological reason(s) behind persistent mother-to-child transmission (MTCT) of HIV (albeit at reduced rate compared to the preantiretroviral therapy era) in spite of the successful implementation of advanced control measures in many African countries remains a priority concern to many HIV/AIDS control programs. This may be partly due to differences in host immunogenetic factors in highly polymorphic regions of the human genome such as those encoding the killer-cell immunoglobulin-like receptor (KIR) molecules which modulate the activities of natural killer cells. The primary aim of this study was to determine the variants of KIR genes that may have a role to play in MTCT in a cohort of infants born to HIV-infected mothers in Yaoundé, Cameroon. We designed a cross-sectional study to molecularly determine the frequencies of 15 KIR genes in 14 HIV-exposed infected (HEI), 39 HIV-exposed/uninfected (HEU), and 27 HIV-unexposed/uninfected (HUU) infants using the sequence specific primer polymerase chain reaction (PCR-SSP) method. We found that all 15 KIR genes were present in our cohort. The frequency of *KIR2DL1* was significantly higher in the unexposed (control) group than in the HIV-exposed group (OR = 0.22, *P* = 0.006). Stratifying analysis by infection status but focusing only on exposed infants revealed that *KIR2DL5*, *KIR2DS1*, and *KIR2DS5* were significantly overrepresented among the HIV-exposed/uninfected compared to infected infants (OR = 0.20, *P* = 0.006). Similarly, the frequencies of *KIR2DS1*, *KIR2DS5*, and *KIR2DL5* were significantly different between infants perinatally infected with HIV (HIV+ by 6 months of age) and HIV-negative infants. Our study demonstrates that *KIR* genes may have differential effects with regard to MTCT of HIV-1.

## 1. Introduction

Infection with HIV can be considered a breach to both the innate and adaptive immune systems [1]. NK cells, a key component of the cellular arm of the innate immune response to invasive pathogens, constitute the front line of defence against viral infections including HIV. They operate through two main mechanisms: cytotoxic destruction (using perforin, granzymes, and tumor necrosis factor (TNF)) and release of cytokines that regulate the adaptive arm of the immune system [2]. NK cells can specifically target stress ligands expressed on HIV-infected host cells leading to the autolysis of the infected cell. Furthermore, secretion of chemokines by NK cells in response to HIV infection can block new cycles of infection [3]. The critical role of NK cells in HIV infection control is also evidenced by the expansion of the NK cell population and the change in distribution of NK cell subsets in acute HIV infection [4]. NK cells represent 2-10% of all the leukocytes in the blood [1]. However, they express a variable repertoire of activating and inhibitory receptors on their cell surface including NKG2D, NKp46, CD94/NKG2A, and killer-cell immunoglobulin-like receptors (KIRs) which are the most characterized and representative of them [5].

KIRs are highly polymorphic and are capable of triggering both activating and inhibitory signals in the carrier NK cell to effect its natural killing function. Such regulation affects both innate and adaptive immunities, particularly with regard to antiviral responses to HIV and many other viruses. To date, 16 KIR genes, 1110 alleles, and more than 50 haplotypes have been identified in different populations (KIR release 2.9.0 of December 2019) [6]. Among them, are seven genes encoding receptors transmitting activating signals through their short cytoplasmic tails (*KIR2DS1*, *KIR2DS2*, *KIR2DS3*, *KIR2DS4*, *KIR2DS5A*, *KIR2DS5B*, and *KIR3DS1*) and eight genes encoding inhibitory receptors with long cytoplasmic tails (*KIR2DL1*, *KIR2DL2*, *KIR2DL3*, *KIR2DL5A*, *KIR2DL5B*, *KIR3DL1*, *KIR3DL2*, and *KIR3DL3*). The exception is with *KIR2DL4* which has both activating and inhibitory functions [7, 8]. Framework genes *KIR3DL3*, *KIR2DL4*, *KIR3DL2*, and *KIR3DP1* are present in almost all human genomes, with rare exceptions [9]. KIR genes are located in 15 different loci on chromosome 19q13.4, and some of them are allelic variants, *KIR3DL1/S1*, *KIR2DL2/L3*, and *KIR2DS3/S5* [10, 11]. Various evolutionary forces namely the response to pathogens has primarily contributed to the diversity in the repertoire of KIR genes ([12], p. 2). The importance of polymorphism in KIR genes in HIV infection has only been reported in a few populations worldwide [13, 14].

Africa is the most affected region by HIV/AIDS in the world, with young women disproportionally affected. In 2018, close to 37.9 million people were living with HIV, of whom 1.7 million were children and adolescents under the age of 15 years, the majority of whom (90%) were in sub-Saharan Africa [15]. In 2018, Cameroon had 23,000 (22,000-41,000) new HIV infections and 18,000 (25,000-33,000) AIDS-related deaths [16]. The estimated number of new paediatric HIV infections was 4500 in 2017, and the vertical transmission rate was 13% in breastfed infants [17]. Before the wide availability of antiretroviral therapy (ART), the majority of HIV-infected infants would die under the age of 2 years with 33% dying before their first birthday [18].

In Cameroon, preventive measures to curb the burden and transmission of HIV/AIDS have been undertaken such as the use of antiretroviral prophylaxis and the Prevention of Mother-to-Child Transmission (PMTCT) program. Despite these, few neonates still acquire the infection. However, a majority of infants do not acquire the infection or remain uninfected despite breastfeeding from mothers who are not virally suppressed and who are not on any ART regimen [19]. A growing number of reports from other parts of the world have demonstrated that susceptibility to HIV infection may be associated with a variety of different host immunogenetic factors such as the KIRs ([20–22], p. 2; [14]). Heterogeneity in frequencies and distribution of KIR genes, genotypes, and haplotypes among populations may explain their role in HIV acquisition. The current study describes the frequencies and distribution of KIR genes, genotypes, and haplotype profiles in a cohort of HIV-exposed infants with the aim to determine their role in mother-to-child transmission of HIV-1 among Cameroonians.

## 2. Materials and Methods

This study was nested within the PREVENT-IT study cohort (CIPHER Pediatric HIV Matters project) which was designed to investigate the impact of *in utero* exposure to Tenofovir on neonatal tubulopathies. We conducted a cross-sectional study in four hospitals in Yaoundé, Cameroon (Green City Hospital, Efoulan, Mbiyem-Assi and CASS Nkoldongo) over a period of one year from April 2018 to May 2019. Recruitment of participants was done through convenience sampling. Sensitization of participants was done in HIV care and maternity units of the various hospitals. Our study population was made up of three groups: HIV-1-positive infants born of HIV-1-positive mothers (exposed-infected: HEI), HIV-1-negative infants born of HIV-1-positive mothers (exposed-uninfected: HEU), and HIV-1-negative infants born of HIV-1-negative mothers (unexposed-uninfected: HUU). Only infants whose parents had given proxy consent were included in the present study. These infants were all of the same age (6 weeks). Mothers of both groups of infants were screened for Hepatitis B Surface Antigen (HBsAg) and Hepatitis C Core Antigen (HCVcAg) using the ELISA technique (QuickTiter, Cell Biolab, INC, San Diego). This study was approved by the Institutional Ethics Committee for Research on Human Health, of the University of Douala, ethical clearance No. 1639IEC-UD/06/2018/T. Administrative authorization was equally obtained from the various collection sites in accordance with the ethical guidelines of the 1975 Declaration of Helsinki.

### 2.1. Clinical Characteristics of Mothers and Children

Clinical information from the mother-child dyads was obtained using a structured questionnaire. These included feeding practices of the infants which can be artificial or breast, the type of delivery documented as full-term vaginal delivery (FTVD) or caesarean section, and mother's ART regimen, weight, and height.

### 2.2. Specimen Collection, Storage, DNA Extraction, and HIV Screening

Six weeks after delivery (mother's visit 1), up to 5 ml of whole blood was collected from both mother and child into ethylenediaminetetraacetic acid (EDTA) anticoagulant tubes. Each sample was processed to isolate the peripheral blood mononuclear cells (PBMCs), buffy coat, and plasma isolated within six hours of collection. PBMCs were stored immediately at -20°C for 2 hours and transferred in -80°C until required for further analysis. Each participant was tested for HIV-1, and their viral load was quantified using the Amplicor HIV-1 DNA PCR assay (Roche Diagnostics, Branchburg, NJ) as previously described [23].

### 2.3. Genotyping of KIR Genes

Genomic DNA was extracted from buffy coat using the QIA amp DNA Blood Mini kit (Qiagen Ltd, Germany) according to the manufacturer's instructions and quantified using the NanoDrop spectrophotometer. KIR genotyping was carried out using sequence specific primer polymerase chain reaction (SSP-PCR) as previously described [24]. The mix was composed of 2 *μ*l of DNA polymerase (PrimeStar GXL, Takara Bio Europe, France), 270 *μ*l of *α*QH2O, 60 *μ*l of 5X buffer, and 9 *μ*l of dNTPs (10 mM) per sample. Briefly, two pairs of sequence specific primers were used to amplify each of 14 functional KIR genes: 2DS1, 2DS2, 2DS3, 2DS4, 2DS5, 2DL1, 2DL2, 2DL3, 2DL4, 2DL5, 3DS1, 3DL1, 3DL2, 3DL3, and the pseudogene 2DP1. Amplicons were electrophoresed in 2% agarose gel and visualized under ultraviolet light for the presence or absence of each gene.

### 2.4. KIR Genotypes and Haplotypes

KIR genotypes were assigned according to the allele frequency net database (http://www.allelefrequencies.net). In the assessment of the KIR genotypes, group B genotypes were defined by the presence of one or more of the following genes: KIR2DL5, KIR2DS1, KIR2DS2, KIR2DS3, KIR2DS5, and KIR3DS1. Conversely, the stable group A genotype was defined by the absence of all the above-mentioned genes and the presence of KIR3DL1, KIR2DL1, KIR2DL3, and KIR2DS4 genes. KIR haplotypes AA (A) and Bx were assigned as previously described [8]. Briefly, individuals carrying *KIR2DL1*, *KIR2DL3*, *KIR2DL4*, *KIR2DS4*, and *KIR3DL1* genes in addition to the framework genes were assigned the AA haplotype. Individuals carrying all AA haplotype genes and any one of the following genes, *KIR2DL2*, *KIR2DL5*, *KIR2DS1*, *KIR2DS2*, *KIR2DS3*, *KIR2DS5*, and *KIR3DS1*, were denoted as AB haplotype while individuals lacking any of the following, *KIR2DL1*, *KIR2DL3*, *KIR3DL1*, and *KIR2DS4*, were BB haplotype. Given the difficulties in distinguishing between AB and BB haplotypes, we coded all AB and BB carriers as Bx [25].

### 2.5. Statistical Analysis

Statistical analyses were done using STATA v16.1. Frequencies of genes, alleles, genotypes, and haplotypes were determined by direct counting. Differences between groups (HUU, HEU, and HEI) were computed using the Kruskal–Wallis test, Chi-squared, or Fisher exact test as may be appropriate. In multivariate models, the Mantel-Haenszel odd ratios were calculated controlling for sex, type of delivery and feeding practice, and adjusted *P* values reported. *P* values < 0.05 were considered significant. Correction for multiplicity testing was performed using the Bonferroni method.

## 3. Results

### 3.1. Clinical and Demographic Details

Genotyping of KIR genes was performed on 80 infants (14 HIV-exposed infected, 39 HIV-exposed/uninfected born to HIV-infected mothers, and 27 HIV-unexposed/uninfected born to HIV-uninfected mothers). All infants were recruited at the same age (six weeks). The population and clinical characteristics of HEI, HEU, and HUU infants are presented in Table 1. The median viral load of mothers of HEI infants was 7511 copies/ml while that of mothers of HEU infants was 466 copies/ml (*P* = 0.012). Breastfeeding was the most represented type of feeding. Female infants were overrepresented in the study population (56.4%).

### 3.2. Comparative Frequencies of KIR Genes in HUU, HEU, and HEI Infants

**Figure 1:**
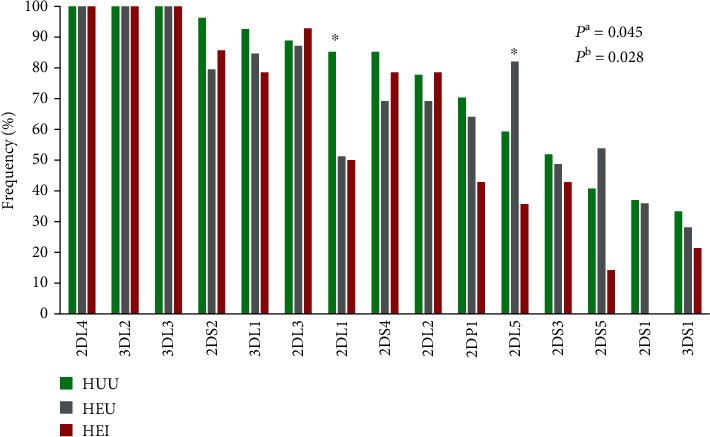
KIR gene frequencies in the study population. *P* is the *P* value from the Kruskal–Wallis test comparing all 3 groups. *P*^a^ is the *P* value of KIR2DL1 and *P*^b^ is the *P* value of KIR2DL5.

We report here the frequencies of 15 KIR genes in an infant Cameroonian cohort (Figure 1). The frequency of individual KIR genes varied from 28.8% to 100.0%. As expected, the framework genes investigated in this study (*KIR3DL3*, *KIR3DL2*, and *KIR2DL4)* were present in all 80 infants. Three inhibitory genes (*KIR2DL2*, *KIR2DL3*, and *KIR3DL1*), and two activating genes (*KIR2DS2* and *KIR2DS4*) were present in >70.0% of the study population. The frequency of *KIR2DL1* was significantly higher in the HIV-unexposed/uninfected group compared to their HIV-exposed counterparts, i.e., infants born to HIV+ mothers (85.2% vs. 50.9%, aOR: 0.22, *P* = 0.006). Interestingly, none of the HEI infants had the activating *KIR2DS1* gene which was present in 14 out of 39 (35.9%) HEU and 10 out of 27 (37.0%) HUU infants (Figure 1).

Comparing infants who acquired HIV-1 through MTCT by 6 weeks of age (*n* = 14) to their uninfected counterparts of similar age (*n* = 66), we found that the inhibitory *KIR2DL5* and two activating *KIR2DS5* and *KIR2DS1* were significantly overrepresented in the uninfected group (Table 2), suggesting that these genes may have a role to play in *in utero* acquisition of HIV-1.

A total of 53 infants were born to HIV-1-infected mothers who were on a combination ART to prevent MTCT of HIV to their babies *in utero*. HIV testing at 6 weeks of age revealed that the majority 39 out of 53 infants were free of HIV-1, but 26.4% (14 out of 53) had acquired the infection. In this group of HIV-exposed infants, we observed that the same group *KIR* genes described above (*KIR2DL5*, *KIR2DS1*, and *KIR2DS5*) were significant overrepresented in the exposed-uninfected group and less frequent in the HEI group (Table 3).

A total of 61 distinct KIR genotypes were found in the study population, 6 of which have not been previously reported in public databases.

## 4. Discussion

Natural killer (NK) cells have the ability to kill virally infected cells without prior sensitisation. They mediate their antiviral activities through a cascade of receptors on their cell surface among which are the killer-cell immunoglobulin-like receptors (KIRs). This study contributes to highlighting further the important role of *KIR* genes in HIV acquisition. To our knowledge and albeit with a small sample size, this is the first report of KIR diversity in HIV-exposed infants in any sub-Saharan Africa region. The aim of the present study was to determine which of the individual *KIR* genes or genotypes may be associated with MTCT in a cohort of infants born to HIV+ mothers in Yaoundé, Cameroon. We recruited 80 infants, 53 of whom were born to HIV-infected mothers in combination with ART and 27 born to HIV-negative mothers as an infant population control group. Of the 53 HIV-exposed infants, 14 (26.4%) tested HIV positive at 6 weeks of age (Table 1). DNA was extracted from each consented participant and used for KIR genotyping as described in “Materials and Methods.” Six activating genes (2DS1, 2DS2, 2DS3, 2DS4, 2DS5, and 3DS1), seven inhibitory genes (2DL1, 2DL2, 2DL3, 2DL5, 3DL1, 3DL2, and 3DL3) in addition to 2DL4 (that has both inhibitory and activating properties), and a pseudogene 2DP1 were investigated in this study population (Figure 1). Many studies have shown that several factors associated with mothers such as viral load and ART uptake can influence the vertical transmission of HIV [26]. However, several other factors related to the child may also be involved in HIV acquisition or resistance. KIR molecules are critical regulators of NK cell effector function and may potentially influence HIV acquisition through NK cell-based innate anti-HIV immune activity [25]. KIR3DS1 and KIR2DS1 were the least frequent in our studied population (Figure 1) and corroborate with findings from other African studies [25, 27]. *KIR2DS1* was absent in the HIV-1-infected group but present in 37.0% of the unexposed/uninfected infants (population control group) (Table 2). This finding is difficult to explain but could be suggestive of a role for *KIR2DS1* in protecting against *in utero* acquisition of HIV-1. But given the small sample size, our finding should be interpreted with caution as the significance only became marginal in multivariate models adjusting for sex, delivery mode, feeding practices, and correction for multiple testing using the Bonferroni technique.

We observed consistently that the frequencies of activating *KIR2DS1*, *KIR2DS5*, and inhibitory *KIR2DL5* genes were higher in the HIV-unexposed/uninfected and the HIV-exposed/uninfected groups compared to infants with perinatally acquired HIV infection (HEI) (Figure 1). Further studies are warranted with larger sample sizes to confirm these findings. *KIR2DS1* was the second least frequent gene (30.0%) in this Cameroonian study population. It has previously been reported to be associated with a low viral load in a Zimbabwean study [25]. We observed that *KIR2DS1* was only present in infants born to mothers with a low viral load (Table 3). This result corroborate with those of an adult study of phenotypic and functional characterization of natural killer cells in antiretroviral naive HIV+ patients in Cameroon [27]. NK activity is often more pronounced in infected individuals than in healthy population, and it is thought that the innate ability to resist viral infection is linked to activating genes [13]. Contrary to these expectations, we observed that inhibitory *KIR2DL1* and *KIR2DL5* genes may also be involved in resisting perinatal acquisition of HIV-1 (Figure 1). This is difficult to explain but could be due in part to a low power of inhibition on the activity of NK cells [28]. However, some conflicting reports from Western populations have implicated the *2DL5* gene with both susceptibility to HIV infection [26] and resistance to hepatitis B infection [29]. KIR genes are highly polymorphic and vary across species, race, and ethnicity [30–32], and its activation might also depend on many other factors [26] that are beyond the scope of the present study.

The extensive variation observed in the KIR loci is the origin of the many genotypes and haplotypes reported to date in public databases [33]. We found that the AA genotype was only present in the infected group (Table S1). Some studies have found similar results with higher AA genotype frequency in infected vs. noninfected individuals [25, 26]. In contrast, one study found the AA genotype only in the uninfected population [13]. Further studies with larger sample sizes are warranted to confirm or refute our findings in this and other populations. Investigating the impact of KIR molecules on the function of NK cells in mediating susceptibility to viral infections is important, and we believe our findings will go a long way to help with better design of future studies in our subregion.

## 5. Conclusion

The present study confirms the diverse nature *KIR* genes in modulating susceptibility or resistance to viral infections focusing on MTCT of HIV-1 even in this small cohort of Cameroonian population. Our findings suggest that certain *KIR* genes may have a role to play in *in utero* and/or perinatal acquisition of HIV-1 irrespective of exposure to combination ART. We found 6 potentially unique KIR genotypes that have not been previously reported.

## Figures and Tables

**Table 1:** Demographic and clinical characteristics of the study participants.

Characteristics		Status	
	HUU, *n* (%)	HEU, *n* (%)	HEI, *n* (%)
Number	27 (33.6)	39 (48.8)	14 (17.5)
Female	15 (55.6)	22 (56.4)	6 (42.9)
Mother parameters			
On ART	—	35 (89.7)	14 (100.0)
Mean log HIV	—	7 (8.5)	6 (9.6)
Delivery			
Normal	25 (92.6)	35 (89.7)	12 (85.7)
Caesarian	2 (7.7)	4 (10.3)	2 (14.3)
Feeding			
Breast	22 (81.5)	30 (76.9)	10 (71.4)
Bottle	4 (14.8)	8 (20.5)	4 (28.6)
Mixed	1 (3.7)	1 (2.6)	0 (0.0)
Other			
Weight in kg, median (IQR)	5.2 (4.8-5.5)	5.0 (4.6-5.7)	5.5 (4.5-6.0)
Height in cm, median (IQR)	55.0 (53.0-56.0)	53.0 (52.0-56.0)	54.0 (53.0-58.0)
BMI	1.8 (1.6-1.9)	1.8 (1.6-1.9)	1.7 (1.5-2.1)

ART, antiretroviral therapy; %, percentage; BMI, body mass index.

**Table 2:** KIR gene frequencies stratified by disease status.

*KIR* gene	HIV status		aOR (95% CI)	*P*
	Negative, *n* (%)	Positive *n* (%)		
Inhibitory				
2DL1	43 (65.2)	7 (50.0)	0.74 (0.22-2.51)	0.632
2DL2	48 (72.7)	11 (78.6)	1.71 (0.38-7.64)	0.478
2DL3	58 (87.9)	13 (92.9)	1.62 (0.13-20.68)	0.705
2DL4	66 (100.0)	14 (100.0)	—	—
2DL5	48 (72.7)	5 (35.7)	0.31 (0.10 - 0.93)	0.028
3DL1	58 (87.9)	11 (78.6)	0.68 (0.20-2.24)	0.521
3DL2	66 (100.0)	14 (100.0)	—	—
3DL3	66 (100.0)	14 (100.0)	—	—
Activating				
2DS1	24 (36.4)	0 (0.0)	—	0.023
2DS2	57 (86.4)	12 (85.7)	1.16 (0.27-5.09)	0.843
2DS3	33 (50.0)	6 (42.9)	0.82 (0.23-2.92)	0.762
2DS4	50 (75.8)	11 (78.6)	1.75 (0.40-7.59)	0.451
2DS5	32 (48.5)	2 (14.3)	0.14 (0.02-1.11)	0.029
3DS1	20 (30.3)	3 (21.4)	0.70 (0.15-3.31)	0.647
Pseudogene				
2DP1	44 (66.7)	14 (100.0)	0.45 (0.12 - 1.71)	0.230

The HIV-negative group comprises 66 infants (27 born to HIV-negative mothers and 39 to HIV+ parents). HIV-positive infants *n* = 14. aOR: the Mantel-Haenszel odds ratio adjusted for sex, type of delivery, and feeding practice. *P*: adjusted *P* values.

**Table 3:** KIR distribution among HIV-exposed infants.

KIR gene	HIV exposed		aOR (95% CI)	*P*
	Uninfected, *n* (%)	Infected *n* (%)		
Inhibitory				
2DL1	20 (51.3)	7 (50.0)	1.18 (0.29-4.75)	0.814
2DL2	27 (69.2)	11 (78.57)	1.89 (0.33-10.68)	0.461
2DL3	34 (87.2)	13 (92.86)	1.78 (0.12-25.44)	0.668
2DL4	39 (100)	14 (100.0)	—	—
2DL5	32 (82.1)	5 (35.7)	0.20 (0.05-0.72)	0.006
3DL1	33 (84.6)	11 (78.6)	0.67 (0.18-2.46)	0.540
3DL2	39 (100.0)	14 (100.0)	—	—
3DL3	39 (100.0)	14 (100.0)	—	—
Activating				
2DS1	14 (35.9)	0 (0.0)	—	0.022
2DS2	31 (79.5)	12 (85.7)	1.77 (0.39-8.10)	0.453
2DS3	19 (48.7)	6 (42.9)	0.89 (0.21-3.84)	0.875
2DS4	27 (69.2)	11 (78.6)	2.05 (0.42-9.98)	0.366
2DS5	21 (53.9)	2 (14.3)	0.06 (0.00-1.09)	0.009
3DS1	11 (28.2)	3 (21.4)	1.04 (0.17-6.41)	0.965
Pseudogene				
2DP1	25 (64.1)	14 (100.0)	0.60 (0.15-2.49)	0.482

Fifty-three (53) infants were born to HIV-infected mothers, 39 were uninfected by 6 weeks of age, and 14 were HIV infected. aOR: the Mantel-Haenszel odds ratio adjusted for sex, type of delivery, and feeding practice. *P*: adjusted *P* values.

## Data Availability

The clinical, biological, and demographic data used to support the findings of this study are included within the article.

## Supplementary Materials

Supplementary Materials Table S1: represents the entire genotypes found in the study population with their frequencies. Common forms represent genotypes already present in others countries of the world, and novel forms are unique in Cameroon. This table represents also different genes composing each genotype and their frequencies in the study population. We have two types of genotype: AA and Bx. Given the difficulties in distinguishing between AB and BB haplotypes, we coded all AB and BB carriers as Bx.
