# Supplementary material for: Killer-Cell Immunoglobulin-Like Receptors (KIR) in HIV-Exposed Infants in Cameroon
Source: J Immunol Res. 2021 Jan 13;2021:9053280. doi: 10.1155/2021/9053280 (PMC7817297; doi:10.1155/2021/9053280)
Supplement: Supplementary Materials — Table S1: represents the entire genotypes found in the study population with their frequencies. Common forms represent genotypes already present in others countries of the world, and novel forms are unique in Cameroon. This table represents also different genes composing each genotype and their frequencies in the study population. We have two types of genotype: AA and Bx. Given the difficulties in distinguishing between AB and BB haplotypes, we coded all AB and BB carriers as Bx. [file 9053280.f1.docx]

S1 Table: KIR genotype frequency distribution in the study population

|  | **GID** | 3DL1 | 2DL1 | 2DL3 | 2DS4 | 2DL2 | 2DL5 | 3DS1 | 2DS1 | 2DS2 | 2DS3 | 2DS5 | 2DL4 | 3DL2 | 3DL3 | 2DP1 | **n** | **%** |
| --- | --- | --- | --- | --- | --- | --- | --- | --- | --- | --- | --- | --- | --- | --- | --- | --- | --- | --- |
| Commun | | | | | | | | | | | | | | | | | | |
| AA | 485 |  |  |  |  |  |  |  |  |  |  |  |  |  |  |  | 1 | 1.25 |
| AA | 305 |  |  |  |  |  |  |  |  |  |  |  |  |  |  |  | 1 | 1.25 |
| Bx | 5 |  |  |  |  |  |  |  |  |  |  |  |  |  |  |  | 2 | 2.50 |
| Bx | 281 |  |  |  |  |  |  |  |  |  |  |  |  |  |  |  | 3 | 3.75 |
| Bx | 03 |  |  |  |  |  |  |  |  |  |  |  |  |  |  |  | 3 | 3.75 |
| Bx | 11 |  |  |  |  |  |  |  |  |  |  |  |  |  |  |  | 2 | 2.50 |
| Bx | 694 |  |  |  |  |  |  |  |  |  |  |  |  |  |  |  | 1 | 1.25 |
| Bx | 654 |  |  |  |  |  |  |  |  |  |  |  |  |  |  |  | 1 | 1.25 |
| Bx | 416 |  |  |  |  |  |  |  |  |  |  |  |  |  |  |  | 1 | 1.25 |
| Bx | 25 |  |  |  |  |  |  |  |  |  |  |  |  |  |  |  | 1 | 1.25 |
| Bx | 413 |  |  |  |  |  |  |  |  |  |  |  |  |  |  |  | 2 | 2.50 |
| Bx | 385 |  |  |  |  |  |  |  |  |  |  |  |  |  |  |  | 5 | 6.25 |
| Bx | 41 |  |  |  |  |  |  |  |  |  |  |  |  |  |  |  | 1 | 1.25 |
| Bx | 415 |  |  |  |  |  |  |  |  |  |  |  |  |  |  |  | 1 | 1.25 |
| Bx | 09 |  |  |  |  |  |  |  |  |  |  |  |  |  |  |  | 1 | 1.25 |
| Bx | 381 |  |  |  |  |  |  |  |  |  |  |  |  |  |  |  | 1 | 1.25 |
| Bx | 269 |  |  |  |  |  |  |  |  |  |  |  |  |  |  |  | 2 | 2.50 |
| Bx | 43 |  |  |  |  |  |  |  |  |  |  |  |  |  |  |  | 1 | 1.25 |
| Bx | 62 |  |  |  |  |  |  |  |  |  |  |  |  |  |  |  | 2 | 2.50 |
| Bx | 342 |  |  |  |  |  |  |  |  |  |  |  |  |  |  |  | 1 | 1.25 |
| Bx | 370 |  |  |  |  |  |  |  |  |  |  |  |  |  |  |  | 1 | 1.25 |
| Bx | 414 |  |  |  |  |  |  |  |  |  |  |  |  |  |  |  | 1 | 1.25 |
| Bx | 562 |  |  |  |  |  |  |  |  |  |  |  |  |  |  |  | 1 | 1.25 |
| Bx | 571 |  |  |  |  |  |  |  |  |  |  |  |  |  |  |  | 2 | 2.50 |
| Bx | 286 |  |  |  |  |  |  |  |  |  |  |  |  |  |  |  | 1 | 1.25 |
| Bx | 487 |  |  |  |  |  |  |  |  |  |  |  |  |  |  |  | 1 | 1.25 |
| Bx | 421 |  |  |  |  |  |  |  |  |  |  |  |  |  |  |  | 1 | 1.25 |
| Bx | 478 |  |  |  |  |  |  |  |  |  |  |  |  |  |  |  | 1 | 1.25 |
| Bx | 391 |  |  |  |  |  |  |  |  |  |  |  |  |  |  |  | 1 | 1.25 |
| Bx | 343 |  |  |  |  |  |  |  |  |  |  |  |  |  |  |  | 1 | 1.25 |
| Bx | 363 |  |  |  |  |  |  |  |  |  |  |  |  |  |  |  | 1 | 1.25 |
| Bx | 104 |  |  |  |  |  |  |  |  |  |  |  |  |  |  |  | 1 | 1.25 |
| Bx | 456 |  |  |  |  |  |  |  |  |  |  |  |  |  |  |  | 1 | 1.25 |
| Bx | 627 |  |  |  |  |  |  |  |  |  |  |  |  |  |  |  | 1 | 1.25 |
| Bx | 361 |  |  |  |  |  |  |  |  |  |  |  |  |  |  |  | 2 | 2.50 |
| Bx | 341 |  |  |  |  |  |  |  |  |  |  |  |  |  |  |  | 1 | 1.25 |
| Bx | 202 |  |  |  |  |  |  |  |  |  |  |  |  |  |  |  | 1 | 1.25 |
| Bx | 296 |  |  |  |  |  |  |  |  |  |  |  |  |  |  |  | 1 | 1.25 |
| Bx | 364 |  |  |  |  |  |  |  |  |  |  |  |  |  |  |  | 1 | 1.25 |
| Bx | 678 |  |  |  |  |  |  |  |  |  |  |  |  |  |  |  | 1 | 1.25 |
| Bx | 313 |  |  |  |  |  |  |  |  |  |  |  |  |  |  |  | 1 | 1.25 |
| Bx | 322 |  |  |  |  |  |  |  |  |  |  |  |  |  |  |  | 1 | 1.25 |
| Bx | 240 |  |  |  |  |  |  |  |  |  |  |  |  |  |  |  | 3 | 3.75 |
| Bx | 449 |  |  |  |  |  |  |  |  |  |  |  |  |  |  |  | 2 | 2.50 |
| Bx | 293 |  |  |  |  |  |  |  |  |  |  |  |  |  |  |  | 1 | 1.25 |
| Bx | 573 |  |  |  |  |  |  |  |  |  |  |  |  |  |  |  | 1 | 1.25 |
| Bx | 176 |  |  |  |  |  |  |  |  |  |  |  |  |  |  |  | 1 | 1.25 |
| Bx | 692 |  |  |  |  |  |  |  |  |  |  |  |  |  |  |  | 1 | 1.25 |
| Bx | 443 |  |  |  |  |  |  |  |  |  |  |  |  |  |  |  | 1 | 1.25 |
| Novel | | | | | | | | | | | | | | | | | | |
| N |  |  |  |  |  |  |  |  |  |  |  |  |  |  |  |  | 1 | 1.25 |
| N |  |  |  |  |  |  |  |  |  |  |  |  |  |  |  |  | 1 | 1.25 |
| N |  |  |  |  |  |  |  |  |  |  |  |  |  |  |  |  | 1 | 1.25 |
| N |  |  |  |  |  |  |  |  |  |  |  |  |  |  |  |  | 1 | 1.25 |
| N |  |  |  |  |  |  |  |  |  |  |  |  |  |  |  |  | 1 | 1.25 |
| N |  |  |  |  |  |  |  |  |  |  |  |  |  |  |  |  | 1 | 1.25 |
| n |  | 69 | 50 | 71 | 61 | 59 | 53 | 23 | 24 | 69 | 39 | 24 | 80 | 80 | 80 | 50 | 80 | 100 |
| gene% |  | 86.3 | 62.5 | 88.8 | 76.3 | 73.8 | 66.3 | 28.8 | 30.0 | 86.3 | 48.8 | 42.5 | 100.0 | 100.0 | 100.0 | 62.5 |  |  |

Filled boxes with grey color represent presence of gene and white boxes represent absence of gene; GID, genotype id; %, percentage of infants; n, total number of each genotype in study population; gene%, gene percentage.
